# Scientometric analysis of neglected tropical disease research in Southeast Asia: insights for integrated disease control strategies

**DOI:** 10.1186/s40794-026-00295-2

**Published:** 2026-03-20

**Authors:** Jerico Bautista Ogaya, Christian Joseph N. Ong, Mohamed Mustaf Ahmed, Roel Nickelson M. Solano, Joey Salinas Ramos, Zhinya Kawa Othman, Omar Abdulkarim Saeed Alhammadi, Adriana Viola Miranda, Olalekan John Okesanya, Shuaibu Saidu Musa, Mohammad Faisal Wardak, Lin Xu, Junjie Huang, M. B. N. Kouwenhoven, Don Eliseo Lucero-Prisno III

**Affiliations:** 1 Department of Medical Technology, Institute of Health Sciences and Nursing, Far Eastern University, Manila, Philippines; 2 Center for University Research, University of Makati, Makati, Philippines; 3 Department of Biology, College of Science, De La Salle University, Manila, Philippines; 4 Research Services Office, Palompon Institute of Technology, Palompon, Leyte, Philippines; 5 Faculty of Medicine and Heath Sciences, SIMAD University, Mogadishu, Somalia; 6 Department of Medical Technology, College of Health Sciences Education, University of Mindanao, Davao City, Philippines; 7 Faculty of Pharmaceutical Sciences, Chulalongkorn University, Bangkok, Thailand; 8 Department of Medical Laboratory, University of Hargeisa, Hargeisa, Somalia; 9 Department of Research, Global Health Focus Asia, Bandung, Indonesia; 10 Department of Public Health and Maritime Transport, Faculty of Medicine, University of Thessaly, Volos, Greece; 11 Department of Medical Laboratory Science, Neuropsychiatric Hospital, Aro, Abeokuta, Nigeria; 12 School of Global Health, Faculty of Medicine, Chulalongkorn University, Bangkok, Thailand; 13 Department of Nursing Science, Ahmadu Bello University, Zaria, Nigeria; 14 Paraclinical Department, Faculty of Medicine, Ghalib University, Herat, Afghanistan; 15 Scientific Research Center, Ghalib University, Herat, Afghanistan; 16 Department of Thoracic Surgery, The First Affiliated Hospital, School of Medicine, Zhejiang University, Hangzhou, Zhejiang China; 17 The Jockey Club School of Public Health and Primary Care, Faculty of Medicine, Chinese University of Hong Kong, Hong Kong SAR, China; 18 Centre for Health Education and Health Promotion, Faculty of Medicine, The Chinese University of Hong Kong, Hong Kong SAR, China; 19 Department of Physics, Xi’an Jiaotong-Liverpool University, Suzhou, China; 20 Department of Global Health and Development, London School of Hygiene and Tropical Medicine, London, UK; 21 Research and Innovation Office, Southern Leyte State University, Sogod, Southern Leyte Philippines; 22 Research and Development and Community Extension, John B. Lacson Maritime University, Iloilo, Philippines

**Keywords:** Neglected tropical diseases, Health equity, Infectious diseases, Health system, Precision public health, One health, Genomic surveillance, Disease mapping, Southeast Asia, ASEAN

## Abstract

**Background:**

Neglected tropical diseases (NTDs) remain a major yet unevenly addressed public health challenge in Southeast Asia, where persistent transmission is closely linked to poverty, environmental vulnerability, and health-system constraints. Understanding the evolution of the regional research landscape is essential for informing integrated disease control strategies and strengthening evidence-based policy responses. This study investigated the scientific landscape of NTD research in Southeast Asia, examining temporal trends, thematic trajectories, and patterns of collaboration using a scientometric approach.

**Methods:**

A retrospective scientometric analysis was conducted using Scopus-indexed publications (1906–2024). Eligible records included English-language original research and review articles with at least one Southeast Asian institutional affiliation. Bibliometric performance indicators and science mapping techniques were applied using Bibliometrix (R) and VOSviewer. Analyses included publication trends, citation impact, collaboration networks, keyword co-occurrence, thematic mapping, and conceptual structure modeling.

**Results:**

A total of 12,119 publications were identified, demonstrating sustained growth (AGR: 5.48%), with marked acceleration after 2000. Research productivity was concentrated in Thailand, Indonesia, and Malaysia, while several lower-income ASEAN member states exhibited minimal indexed output. International collaboration networks were dense but asymmetrical, with strong linkages to the United States, United Kingdom, Japan, and Australia, and comparatively limited intra-ASEAN integration. Thematic analyses identified dengue as the dominant motor theme, supported by strong vector biology and molecular epidemiology clusters. Helminthic and zoonotic diseases occupied more peripheral positions. Emerging domains included spatial epidemiology, genomic surveillance, and climate-linked modeling, though One Health integration remained structurally underdeveloped.

**Conclusion:**

Findings delineate a maturing yet structurally bifurcated NTD research ecosystem in Southeast Asia. Strengthening intra-regional scientific networks, diversifying funding architectures, and promoting cross-disciplinary integration will be critical to aligning research production with regional disease burdens and advancing equitable progress toward the WHO 2030 NTD Roadmap.

**Clinical trial registration:**

Not applicable.

## Introduction

Neglected tropical diseases (NTDs) encompass a heterogeneous group of parasitic, viral, bacterial, and fungal infections that disproportionately impact socioeconomically marginalized populations [1]. These diseases persist at the nexus of structural inequities, environmental vulnerabilities, and fragile health systems that affect more than one billion people worldwide [1, 2]. While initiatives such as the WHO NTD Roadmap 2021–2030 have intensified global commitments to elimination and control, progress remains uneven due to variable political prioritization, resource constraints, and persistent challenges in translating research into effective public health action [3, 4].

Southeast Asia (SEA) represents a critical geographic hotspot for the transmission of multiple NTDs, where climatic patterns, dense urbanization, population mobility, persistent rural poverty, and inadequate sanitation interact to sustain high endemicity [5]. These conditions facilitate widespread transmission of dengue, lymphatic filariasis, schistosomiasis, soil-transmitted helminthiases, leptospirosis, and other NTDs that collectively impose a substantial public health burden [5–7]. While several countries have implemented national NTD control and elimination programs, disparities in research capacity, funding allocation, and scientific output remain evident.

Scientometric analysis offers a systematic, quantitative approach to mapping research production, collaboration networks, and thematic evolution that complements traditional scoping and systematic reviews. While reviews synthesize evidence on intervention effectiveness, scientometrics enables the identification of structural research gaps, inequities in knowledge production, and misalignment between disease burden and research investment, which are critical considerations for priority-setting and capacity building in neglected tropical disease control. Previous bibliometric studies on neglected tropical diseases have largely focused on global trends or single diseases, with limited attention to Southeast Asia as a distinct epidemiological and research ecosystem. Moreover, disease-specific publication patterns, regional collaboration structures, and their implications for strategic research prioritization remain underexplored.

Existing scientometric studies largely focus on global research patterns or single diseases such as schistosomiasis, leishmaniasis, or dengue, offering limited insight into Southeast Asia’s collective research landscape [8–13]. This geographical gap is concerning, given the region’s shared epidemiological and socioeconomic determinants and its importance in meeting global NTD elimination milestones. Parallel to these persistent challenges, countries in the region have increasingly expressed commitments to strengthening their public health and research infrastructures. The ASEAN Post-2015 Health Development Agenda emphasizes communicable diseases, NTDs, and emerging threats as priority areas requiring coordinated regional action [8]. In addition, the push for establishing regional disease surveillance networks and integrated research platforms has gained momentum, prompted by lessons from COVID-19 and ongoing vector-borne disease epidemics [9, 10].

As global health shifts toward precision public health, there is a compelling need to understand research systems in Southeast Asia for the integration of genomics, digital technologies, spatial analytics, and environmental modeling to optimize interventions [11, 12]. However, the progress in this scientific transition remains insufficiently characterized in the region. The absence of a comprehensive scientometric assessment constrains policymakers, funding agencies, and scientific communities from identifying knowledge gaps, emerging research domains, and opportunities for enhanced regional collaboration [14, 15]. This study addresses this gap through a comprehensive scientometric analysis of NTD research originating from Southeast Asia. Specifically, it examines longitudinal publication trends, patterns of international collaboration, leading authors, institutions, fundingand journals, as well as the intellectual and conceptual structure of the field through co-occurrence networks, thematic evolution, and factorial analyses. By providing an integrated overview of research productivity, collaboration dynamics, and thematic development, this study aims to provide an evidence base to inform strategic research investment, strengthen regional research capacity, and support the advancement of integrated disease control strategies in Southeast Asia.

## Methods

### Study design

This study employed a retrospective scientometric design to systematically characterize the structural development, collaborative architecture, and thematic evolution of neglected tropical disease (NTD) research in Southeast Asia. Scientometric methodology integrates quantitative performance indicators with network-based science mapping techniques to examine the intellectual, social, and conceptual organization of a research field. By combining bibliographic performance analysis with co-authorship, keyword co-occurrence, and thematic evolution modeling, this approach enables the identification of growth trajectories, collaboration intensity, knowledge consolidation, and emergent research fronts over time. The analytical framework was designed to capture both macro-level productivity trends and meso-level structural dynamics within the regional research ecosystem.

NTDs were operationally defined using an expanded World Health Organization–informed framework encompassing parasitic, bacterial, viral, and zoonotic infections that disproportionately affect marginalized populations and require sustained public health intervention. While certain diseases such as dengue and rabies are not universally classified as NTDs across all global frameworks, their inclusion reflects their sustained epidemiological burden, programmatic prioritization, and integration into regional control strategies within Southeast Asia. The influence of this expanded definition on thematic prominence, particularly the dominance of arboviral research, is explicitly considered in the interpretation of findings. To enhance construct validity and epidemiological alignment, diseases not endemic to Southeast Asia, such as human African trypanosomiasis, dracunculiasis (Guinea worm disease), and onchocerciasis, were deliberately excluded from the search strategy. This exclusion prevents distortion of thematic clustering by non-regional disease domains and ensures that the analytical corpus reflects ASEAN-relevant public health priorities.

### Data source

This study utilized Scopus (Elsevier) as the primary bibliographic database due to its extensive coverage of biomedical and public health journals, structured metadata architecture, and established utility in scientometric investigations. Compared with Web of Science, Scopus offers a broader representation of regional and applied public health journals, a feature particularly relevant for mapping the heterogeneous, policy-oriented NTD research landscape in Southeast Asia. The use of Scopus as the sole bibliographic source ensures high metadata uniformity and significantly reduces the risk of duplicate records across the longitudinal dataset. While multi-database retrieval can marginally increase coverage, it frequently introduces substantial inconsistencies in citation indexing and metadata heterogeneity, specifically regarding author naming conventions, institutional affiliations, and keyword standardization. Such discrepancies require extensive post-hoc reconciliation and can introduce systematic bias through imperfect record matching or fragmented citation counts across disparate platforms. By restricting retrieval to a single, comprehensive database, this study maintains the internal consistency and standardized bibliometric fields—including authorship, institutional/country affiliations, and citation counts, necessary for reliable network construction and reproducible citation-based indicators. This approach aligns with established scientometric practices where the primary objective is structural mapping and relational analysis rather than the exhaustive synthesis typical of a systematic review. Furthermore, Scopus facilitated the export of granular metadata, such as subject classifications and CiteScore indicators, used here to contextualize source influence. It is acknowledged, however, that reliance on a single database may underrepresent non-indexed or locally disseminated research outputs, particularly from lower-income ASEAN member states; consequently, observed disparities in publication volume should be interpreted as differences in indexed scholarly visibility rather than absolute research activity.

### Search strategy and data extraction

**Fig. 1:**
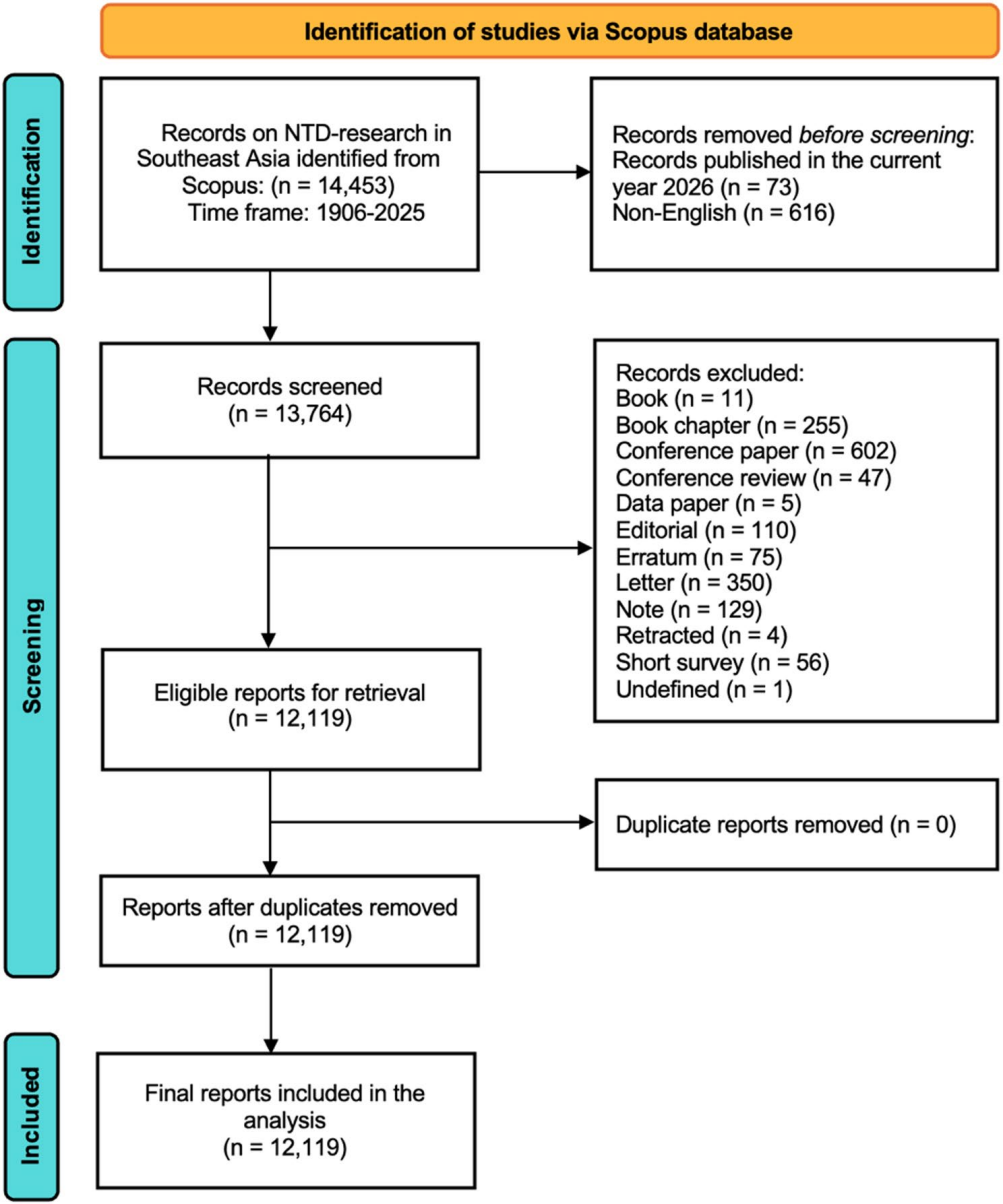
Summary of methods used in the study (adopted from PRISMA Flow Chart)

A structured search strategy was developed to maximize retrieval sensitivity while preserving epidemiological specificity and regional relevance. The search was executed in Scopus on 09 February 2026 and applied to the TITLE-ABS-KEY fields to capture publications in which validated NTD-related terms and Southeast Asian geographic identifiers appeared in titles, abstracts, or author/indexed keywords (Fig. 1).

The query incorporated standardized disease nomenclature and recognized synonyms for regionally endemic NTDs, including dengue, chikungunya, leishmaniasis, lymphatic filariasis, schistosomiasis, soil-transmitted helminthiases, echinococcosis, trachoma, yaws, snakebite envenoming, leprosy, rabies, and mycetoma (Appendix I). Diseases not endemic or epidemiologically relevant to Southeast Asia, such as trypanosomiasis, dracunculiasis, and onchocerciasis, were deliberately excluded to ensure construct validity and alignment with ASEAN public health priorities.

To enhance data stability and citation reliability, publications indexed in 2026 were excluded due to incomplete accrual and indexing variability at the time of retrieval. Eligibility was restricted to peer-reviewed original research articles and review articles published in English-language to ensure comparability of bibliometric indicators and analytical consistency. Records were required to include at least one institutional affiliation from the eleven ASEAN member states: Brunei Darussalam, Cambodia, Indonesia, Lao PDR, Malaysia, Myanmar, the Philippines, Singapore, Thailand, Viet Nam, or Timor-Leste. Editorials, letters, conference proceedings, notes, book chapters, and other non-research document types were excluded to maintain homogeneity in productivity and citation analyses. The search strategy prioritized inclusivity across heterogeneous NTD categories to enable ecosystem-level mapping of the regional research landscape. Although this approach may accentuate high-output disease domains, particularly dengue, such inclusiveness was methodologically justified to preserve system-wide structural representation rather than constrain analysis to disease-specific productivity. Eligible records were exported in CSV format with complete bibliographic metadata for subsequent preprocessing and analysis. Journal-level CiteScore metrics were retrieved directly from Scopus to contextualize source influence within the publication ecosystem.

### Data cleaning and harmonization

To ensure data accuracy and validity, a manual data cleaning was conducted in the extracted dataset. The dataset was cleaned through deduplication of records, standardization of author and institutional names, and manual harmonization of keywords using a curated thesaurus. Minimum thresholds were applied for network analyses (≥ 5 keyword occurrences and ≥ 10 institutional publications) to reduce noise and improve interpretability. It also involved the removal of keyword artifacts and harmonization of terms through a manually curated synonym file (.txt), enabling consistent keyword merging and improved accuracy of analyses. In addition, manual screening of titles and abstracts was conducted to remove irrelevant records (e.g., studies unrelated to NTDs or Southeast Asia despite keyword overlap) to address database noise issues. This step ensured thematic relevance and regional specificity.

### Data analysis and tools

Bibliometric performance and science mapping analyses were conducted using the Bibliometrix/Biblioshiny package in R and VOSviewer (version 1.6.20; Centre for Science and Technology Studies, Leiden University). Performance analysis included computation of annual publication output, citation trajectories, source productivity, institutional output, and highly cited documents. Conceptual structure was explored using multiple correspondence analysis and multidimensional scaling to identify latent thematic groupings and intellectual proximities among keywords. Co-authorship networks were constructed at institutional and country levels to assess collaboration density, connectivity patterns, and structural centrality within the regional research ecosystem. Co-occurrence networks of keywords derived from titles and abstracts were generated using association strength normalization to detect thematic clusters and conceptual interrelationships. Temporal overlay visualization was applied to examine shifts in keyword prominence and to identify emergent research domains across defined time periods. Together, this integrated analytical framework provides a multidimensional and structurally grounded assessment of productivity patterns, collaborative configurations, and thematic evolution within Southeast Asia’s NTD research landscape.

## Results

### Research trend and temporal dynamics

**Fig. 2:**
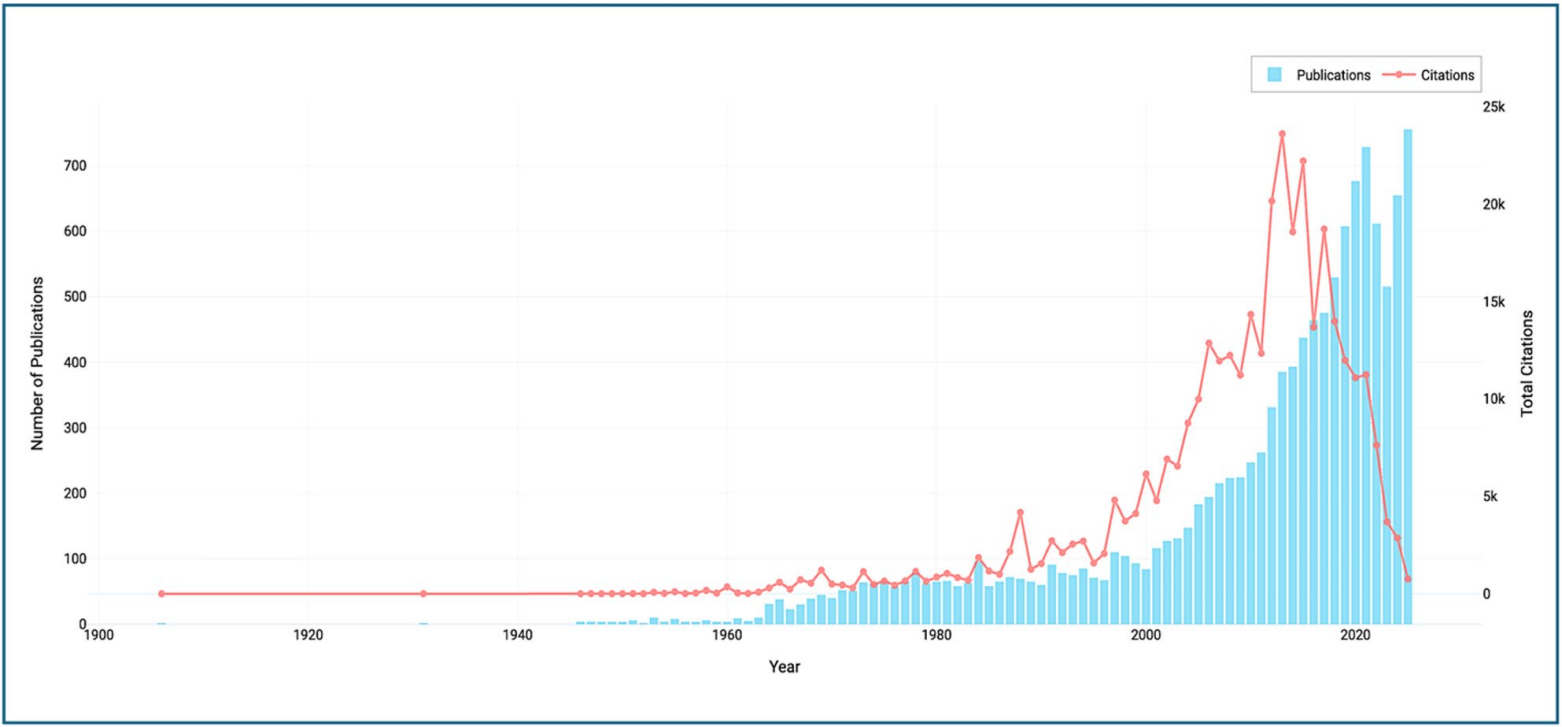
Publication volume trend with annual citation counts for NTD research in Southeast Asia

A total of 12,119 scientific articles on neglected tropical diseases in Southeast Asia, published between 1906 and 2024, were retrieved and analyzed for this study (Fig. 2). The analysis on the temporal trajectory of this publication trend revealed a relatively modest output during the early decades of the 20th century, followed by a progressive acceleration beginning in the early 2000s. The annual publication growth rate was estimated at 5.48%, while the average citation impact reached 27.8 citations per article, indicating moderate yet meaningful global uptake of Southeast Asian research.

The acceleration in publication output after the early 2000s coincides temporally with several structural drivers, including intensified regional dengue epidemics, expansion of WHO-led neglected tropical disease initiatives following the 2012 London Declaration, and increased investment in genomic and vector-borne disease research infrastructure. The post-2015 growth phase further parallels the integration of Southeast Asian datasets into large-scale Global Burden of Disease (GBD) modeling exercises, while thematic diversification after 2020 reflects both the COVID-19 pandemic’s disruption of infectious disease research priorities and increased interest in climate-sensitive disease modeling. These contextual drivers suggest that publication growth is not solely an endogenous maturation process but is shaped by global policy milestones and outbreak-driven funding cycles.

**Fig. 3:**
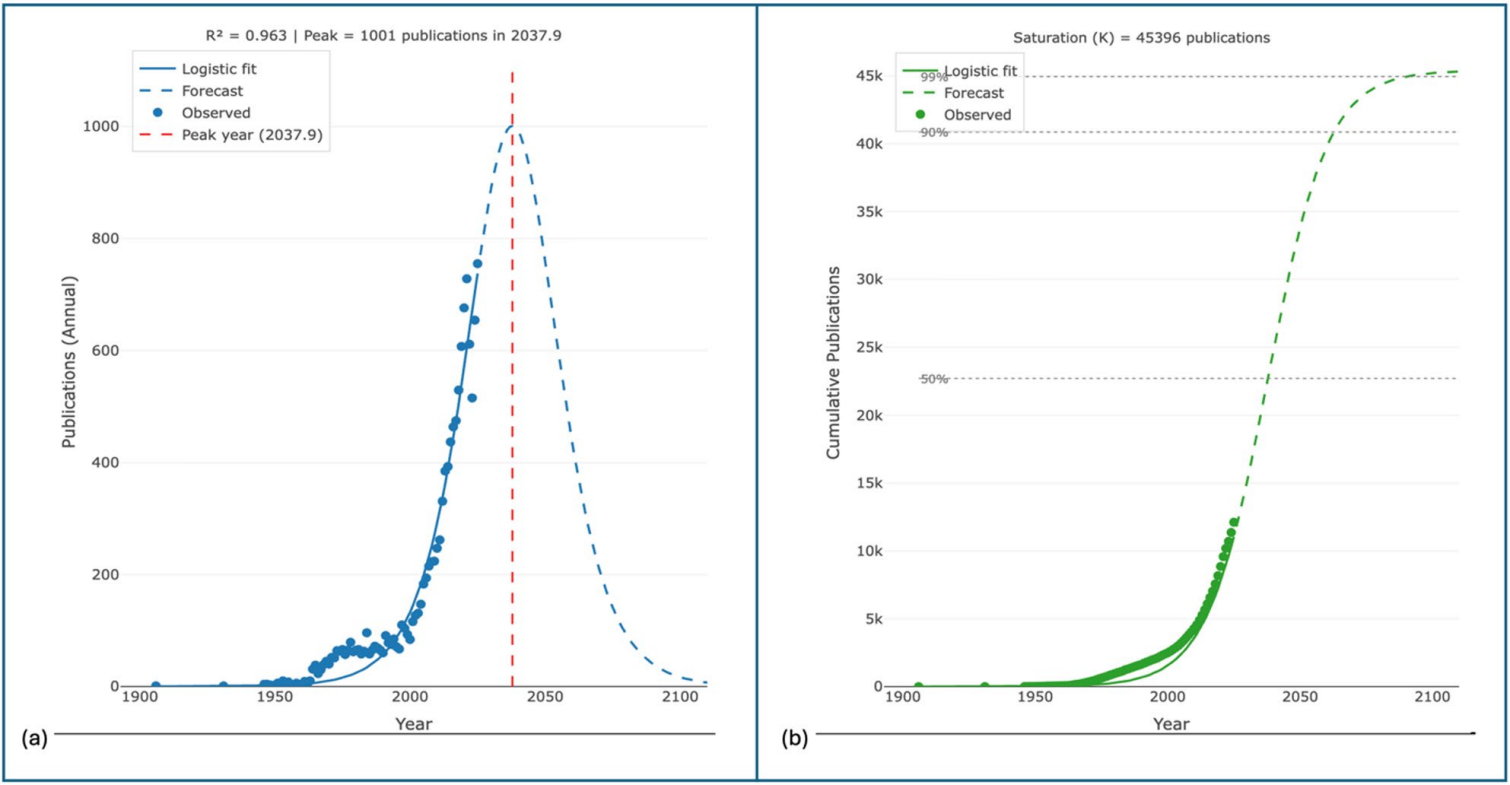
(**a**) Scientific publication life cycle and (**b**) cumulative growth trajectory of NTD research in Southeast Asia

The logistic growth model (Fig. 3a) demonstrated excellent fit (R² = 0.961), indicating that cumulative NTD-related publications in Southeast Asia follow a sigmoidal growth pattern characteristic of maturing research fields. In this study, the model is employed as a descriptive analytical tool rather than a predictive framework. The estimated inflection point around 2033 and the theoretical carrying capacity of approximately 29,105 publications (Fig. 3b) should therefore be interpreted as model-dependent parameters that summarize historical publication dynamics under assumptions of growth saturation.

The observed moderation in growth rates may reflect a transition from rapid output expansion toward greater thematic differentiation, methodological refinement, and translational orientation. The prolonged transition period (delta_t ≈ 45 years) highlights the protracted evolution of NTD research in a region marked by shifting epidemiological pressures and substantial heterogeneity in national research capacities, with significant implications for research investment strategies, capacity-building efforts, and the alignment of scientific production with evolving regional disease-control priorities.

### Most productive countries

**Fig. 4:**
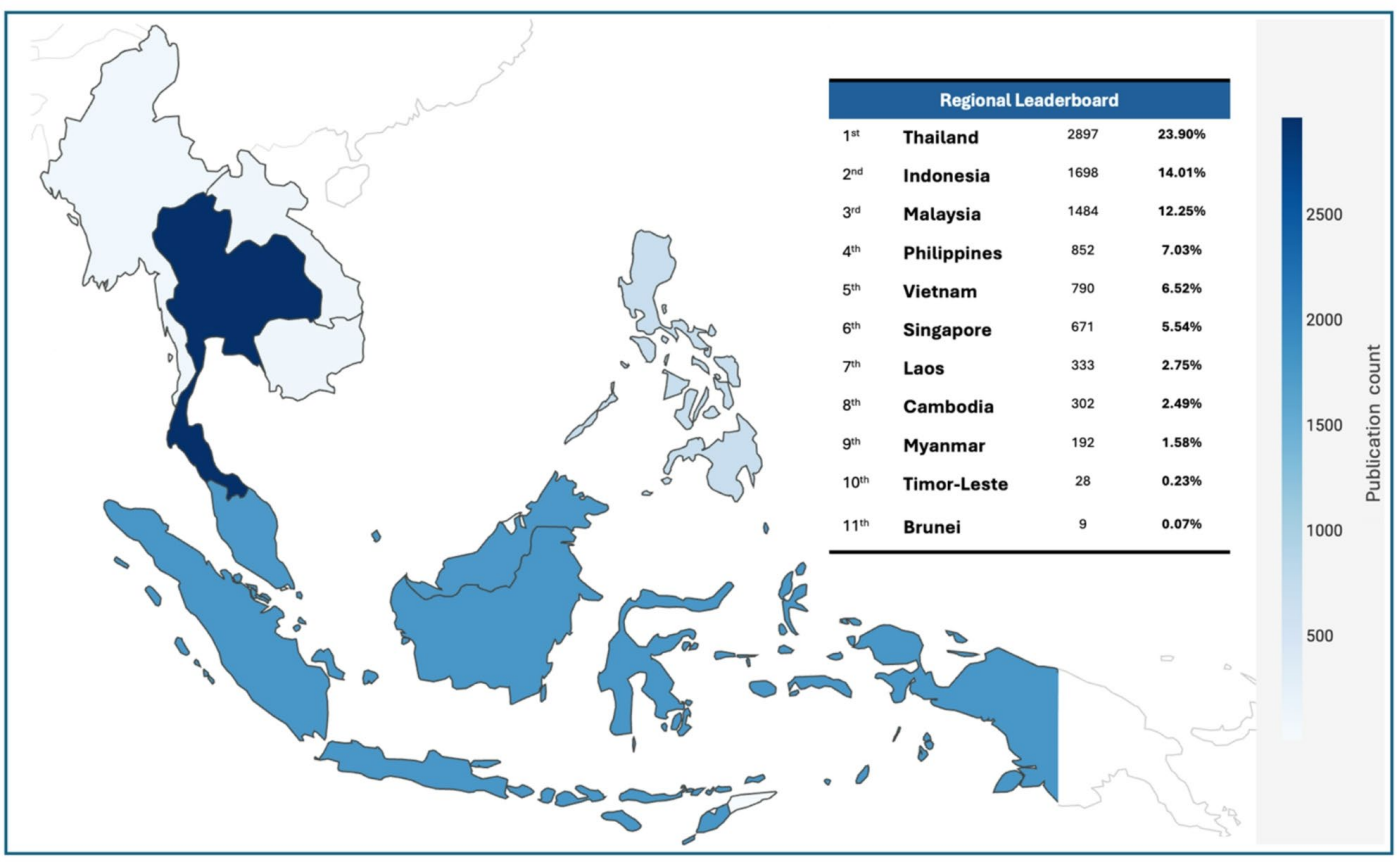
Geographic distribution of NTD publications by country in Southeast Asia (1906–2025)

The regional analysis of research productivity showed marked disparities across Southeast Asia (Fig. 4), with Thailand producing the highest output (23.90%), reflecting its strong research infrastructure and longstanding investment in NTD-related scholarship. Indonesia (14.01%) and Malaysia (12.25%) followed as major contributors, indicating expanding national research capacities supported by growing institutional networks. Mid-tier productivity was observed in the Philippines (7.03%) and Vietnam (6.52%), which demonstrated steady but comparatively modest engagement in the field, while Singapore (5.54%) contributed a smaller volume yet remained notable given its size and high-impact research profile. In contrast, markedly lower outputs from Laos (2.75%), Cambodia (2.49%), Myanmar (1.58%), Timor-Leste (0.23%), and Brunei (0.07%) highlight persistent structural inequities in research funding, institutional capacity, and workforce development. These disparities reveal a region where scientific productivity remains heavily concentrated among a few research-intensive nations, with limited contributions from resource-constrained contexts, thereby shaping the current regional knowledge production and potentially influencing the direction of collaborative research agendas.

When output is normalized against population size and economic capacity, the asymmetry within the ASEAN region becomes even more pronounced. Singapore demonstrates a disproportionately high research intensity and citation influence, illustrating how aggressive GDP-linked R&D investment drives disproportionate visibility in global scientific databases. Conversely, populous nations such as Indonesia and the Philippines exhibit a significant productivity-to-burden deficit, where the volume of indexed publications fails to match the scale of their national disease burdens. This trend reveals a “visibility paradox” in lower-output nations like Laos and Timor-Leste, where minimal indexed productivity likely reflects underrepresentation in international databases and the prevalence of “grey literature” rather than a true absence of indigenous research activity. Such disparities underscore a critical need for regionally coordinated capacity-building initiatives. To ensure an equitable health strategy, the ASEAN research agenda must transition from a model of knowledge transfer to one of horizontal co-production, ensuring that disease elimination efforts are informed by data from all endemic settings.

### Co-authorship and collaborations

**Fig. 5:**
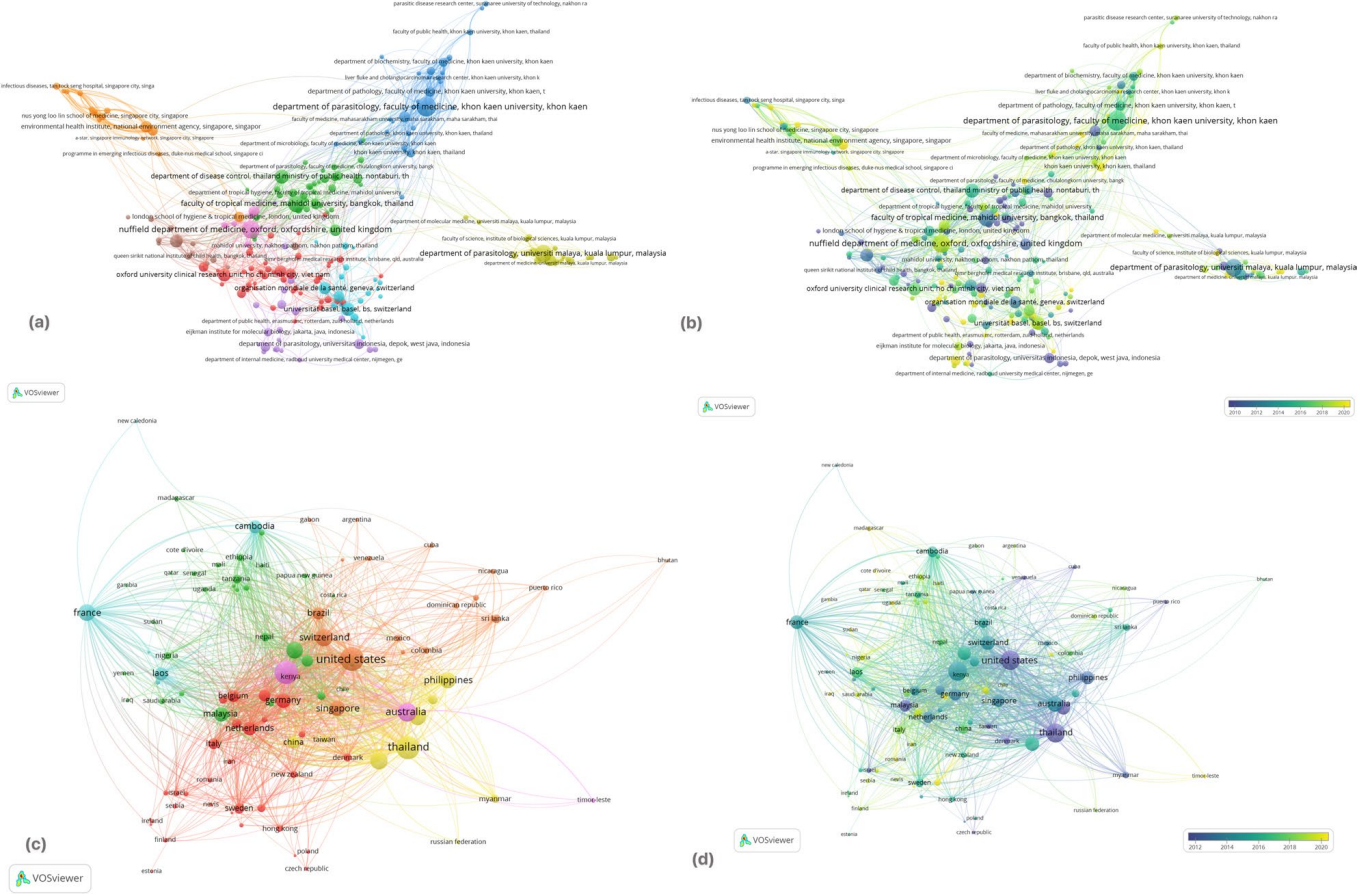
Co-authorship in NTD research among (**a**) institutions through network analysis and (**b**) overlay visualization; and countries collaboration through (**c**) network analysis and (**d**) overlay visualization

The institutional co-authorship analysis reveals a highly centralized network architecture, dominated by a “triad” of high-output research hubs. As illustrated in Fig. 5(a), Mahidol University (Thailand), Khon Kaen University (Thailand), and the University of Oxford’s Nuffield Department of Medicine (UK) emerge as the primary gravitational centers of the network, characterized by the largest node sizes and the highest degree of betweenness centrality. These institutions function as critical knowledge brokers, bridging specialized regional clusters, such as the Southeast Asian parasitic disease groups, with global infectious disease networks. The overlay visualization in Fig. 5(b) further elucidates the evolution of these partnerships; while the foundational nodes in Oxford and Bangkok exhibit cooler tones (indicating long-standing established activity), there is a notable “yellowing” of the network periphery. This temporal shift signifies the recent integration of smaller, specialized research institutes in lower-income endemic regions, suggesting a gradual expansion of the collaborative frontier beyond traditional elite academic centers.

The country-level collaboration networks (Fig. 5c and d) demonstrate a highly interconnected research landscape in which Southeast Asian institutions frequently collaborate with partners in North America, Europe, and East Asia. These partnerships appear to play an important role in facilitating knowledge exchange, access to specialized technologies, and joint publication outputs. At the same time, intra-regional collaboration among ASEAN member states appears comparatively less dense in the co-authorship network. While the increasing participation of institutions from endemic countries suggests a gradual diversification of contributors to the field, the present bibliometric analysis cannot determine the extent to which these patterns reflect shifts in research leadership or agenda setting. Nonetheless, the findings highlight opportunities for strengthening intra-ASEAN scientific collaboration to support regionally coordinated research initiatives.

### Top funding agencies

**Fig. 6:**
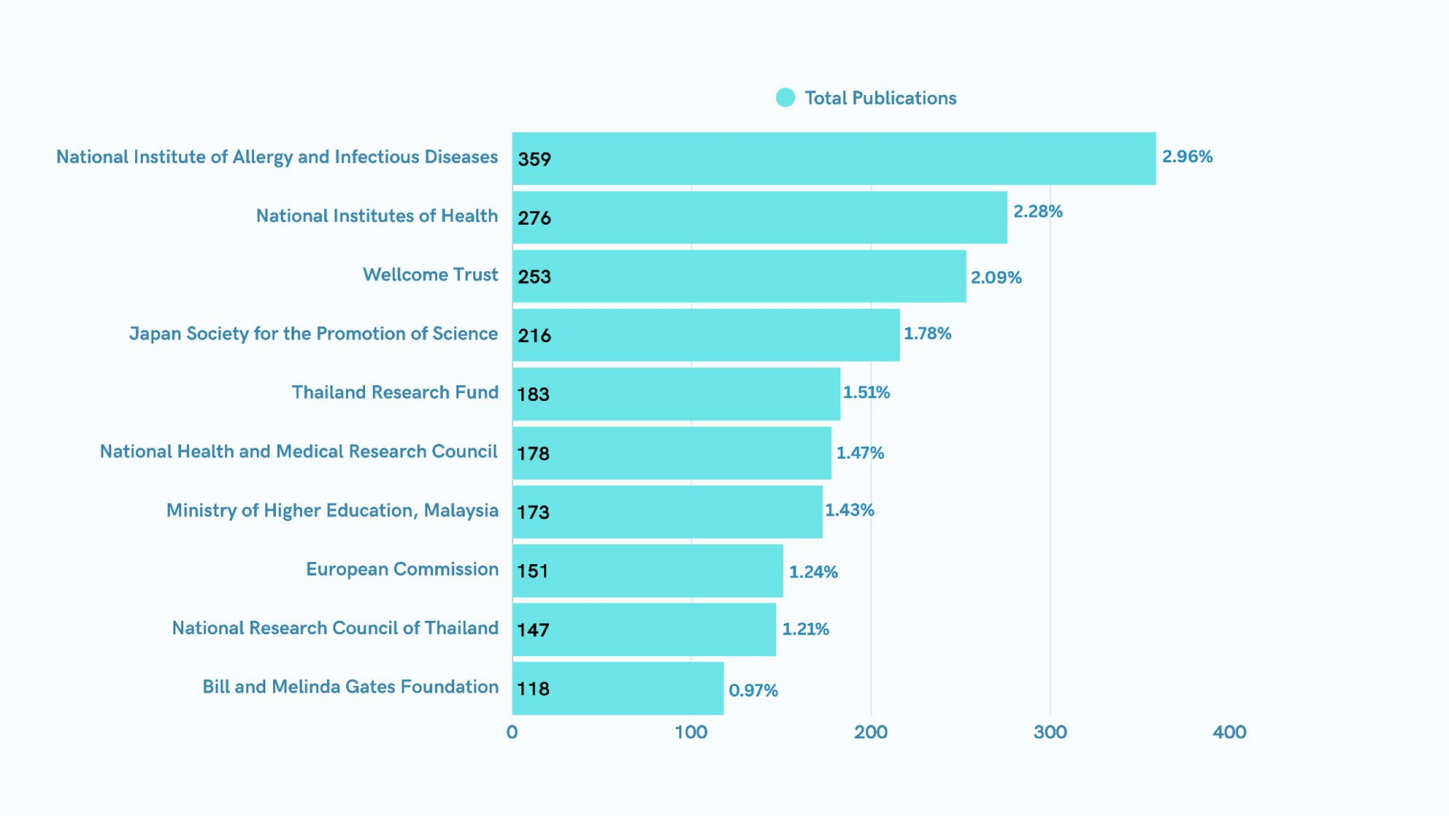
Top 10 most acknowledged funding agencies supporting NTD research in Southeast Asia

The funding landscape for neglected tropical disease research in Southeast Asia reflects a substantial contribution from international funding agencies, particularly the U.S. National Institutes of Health (NIH), the Wellcome Trust, and the Bill & Melinda Gates Foundation (Fig. 6). Additional support from the Japan Society for the Promotion of Science (JSPS) and Australia’s National Health and Medical Research Council (NHMRC) further illustrates the strong participation of extra-regional funders in supporting research activities across the region. These funding patterns appear to coincide with extensive international collaboration networks and the high visibility of certain disease domains, particularly dengue and vector-borne infections.

Domestic funding contributions are also visible, particularly from research-intensive ASEAN member states such as Thailand, Malaysia, and Singapore. National agencies including the Thailand Research Fund and the Malaysian Ministry of Higher Education have supported institutional research programs that contribute to the region’s publication output. However, funding acknowledgments from several lower-income ASEAN countries appear less frequently in the indexed literature, which may reflect differences in national research capacity, funding availability, or publication indexing patterns. These observations highlight potential disparities in the distribution of financial resources supporting NTD research across Southeast Asia, although the present analysis cannot determine causal relationships between funding sources and research agenda setting.

### Most relevant authors

Table 1 highlights the concentration of intellectual leadership in neglected tropical disease research within a few key institutional hubs in Southeast Asia, particularly in Thailand. The prominence of Sithithaworn and Sripa from Khon Kaen University reflects the institution’s longstanding engagement in parasitology and hepatobiliary disease research, areas closely linked to endemic infections in the Mekong subregion. While Sithithaworn ranks first in total publications, the citation indicators suggest a different pattern of influence. For example, Nisalak from the Armed Forces Research Institute of Medical Sciences demonstrates the highest citation impact, with an average of 120.44 citations per paper and the highest h-index among the listed authors. These differences illustrate how productivity and citation influence may reflect distinct roles within the research ecosystem, including sustained regional research output and participation in widely cited international studies.

**Table 1:** Top 10 most productive authors in NTD research in Southeast Asia

Rank	Author’s name	Affiliation	TP (%)	TC	ACP	h-index
1st	Sithithaworn, P.	Khon Kaen University (Thailand)	153 (1.26%)	6,529	42.67	41
2nd	Sripa, B.	Khon Kaen University (Thailand)	117 (0.97%)	8,220	70.26	38
3rd	Nisalak, A.	Armed Forces Research Institute of Medical Sciences (Thailand)	104 (0.86%)	12,526	120.44	54
4th	Ng, L.C.	National Environment Agency (Singapore)	98 (0.81%)	5,739	58.56	42
5th	McManus, D.P.	QIMR Berghofer Medical Research Institute (Australia)	87 (0.72%)	4,720	54.25	35
6th	Odermatt, P.	Universität Basel (Switzerland)	86 (0.71%)	3,706	43.09	35
7th	Leo, Y.S.	Tan Tock Seng Hospital (Singapore)	75 (0.62%)	3,770	50.27	32
8th	Olveda, R.M.	Research Institute for Tropical Medicine (Philippines)	74 (0.61%)	2,891	39.07	35
9th	Sayasone, S.	Ministry of Health, Lao PDR (Laos)	71 (0.59%)	2,072	29.18	27
10th	Lim, Y.A.L.	Universiti Malaya (Malaysia)	70 (0.58)	2,137	30.53	24

*Abbreviations: ACP, average citations per paper; TC, total citations; TP, total publications, Rank was based on TP

The authorship profile also reflects the international nature of NTD research in Southeast Asia. The presence of researchers affiliated with institutions in Australia and Switzerland indicates the continued involvement of extra-regional collaborators in the field. At the same time, the representation of authors from Singapore, Malaysia, the Philippines, and Lao PDR suggests increasing contributions from institutions within the region itself. Although bibliometric data cannot determine the specific dynamics underlying these collaborations, the distribution of authors across institutions highlights a networked research landscape in which both regional and international partners contribute to the development of NTD scholarship in Southeast Asia.

### Most relevant sources

The analysis of publication venues reveals a distinct divergence between regional output volume and global citation influence (Table 2). The Southeast Asian Journal of Tropical Medicine and Public Health (Thailand) serves as the primary repository for regional data, accounting for the highest total publications (7.37% TP), yet it maintains the lowest CiteScore (0.3) among the top ten outlets. Conversely, Western-headquartered journals, specifically PLoS Neglected Tropical Diseases and the American Journal of Tropical Medicine and Hygiene, dominate the impact landscape. Despite lower publication volumes, these international titles achieved significantly higher ACP—46.08 and 44.87, respectively—and commanded the highest h-indices (86 and 71). This suggests that while local journals are essential for regional documentation, research disseminated through Global North publishers achieves substantially greater academic reach and integration into the global scientific discourse.

**Table 2:** Top 10 scopus-indexed journals publishing NTD research in Southeast Asia

Rank	Journal Title	CiteScore 2024	Publisher (HQ)		TP (%)	TC	ACP	h-index
1st	Southeast Asian Journal of Tropical Medicine and Public Health	0.3	SEAMEO TROPMED Network (Thailand)		893 (7.37%)	13,266	14.86	48
2nd	PLoS Neglected Tropical Diseases	7.0	Public Library of Science (USA)		626 (5.17%)	28,845	46.08	86
3rd	American Journal of Tropical Medicine and Hygiene	4.1	American Society of Tropical Medicine and Hygiene (USA)		479 (3.95%)	21,494	44.87	71
4th	PLoS ONE	5.4	Public Library of Science (USA)		241 (1.99%)	12,127	50.32	48
5th	Acta Tropica	4.8	Elsevier (The Netherlands)		233 (1.92%)	3,301	14.16	42
6th	Transactions of the Royal Society of Tropical Medicine and Hygiene	4.9	Oxford University Press (UK)		191 (1.58%)	6,011	31.47	46
7th	Parasites and Vectors	6.1	BioMed Central it(UK)		172 (1.42%)	5,445	31.66	43
8th	Tropical Biomedicine	1.8	Malaysian Society of Parasitology and Tropical Medicine (Malaysia)		149 (1.23%)	2,080	13.96	28
9th	Leprosy Review	1.1	Lepra (UK)		112 (0.92%)	1,179	10.53	19
10th	Journal of the Medical Association of Thailand	0.4	Medical Association of Thailand (Thailand)		109 (0.90%)	1,283	11.77	20
10th	Parasitology Research	3.8	Springer Nature (Germany)		109 (0.90%)	2,461	22.58	29

*Abbreviations: ACP, average citations per paper; TC, total citations; TP, total publications, Rank was based on TP

Geographical distribution of publishers further highlights a reliance on international infrastructure, with 72% of the top-ranked journals based in the USA, UK, or Europe. High-efficiency outlets like PLoS ONE demonstrate the highest ACP (50.32), indicating that multidisciplinary, open-access platforms are highly effective for maximizing the visibility of Southeast Asian NTD research. In contrast, specialized regional journals published in Thailand and Malaysia exhibit lower citation metrics, reflecting a “niche” impact profile characterized by localized relevance rather than broad international citation. This disparity underscores a strategic preference among researchers to target high-impact Western journals for landmark findings while utilizing regional platforms for foundational or surveillance-heavy studies.

### Most influential documents

 The most influential documents reveal that the citation landscape for NTD research in Southeast Asia is dominated by large-scale epidemiological frameworks and global health metrics (Table 3). A significant proportion of the top-cited works (40%) are products of the Global Burden of Disease (GBD) study, published primarily in *The Lancet*. These documents exhibit the highest Normalized Total Citations (NTC), with Naghavi et al. (2015) reaching an NTC of 129.42. This concentration suggests that research achieving the highest academic visibility in the region is that which contextualizes localized NTD data, such as mortality and Disability-Adjusted Life Years (DALYs), within a broader, comparative global health architecture. The analysis indicates that Dengue remains the primary focus of highly influential research, represented by 30% of the top ten list. The study by Bhatt et al. (2013) in *Nature* emerged as the most cited document overall (TC = 7,718), maintaining an extraordinary citation velocity of 551.29 citations per year. The longevity of this influence is further evidenced by the inclusion of Halstead (1988) in *Science*, which remains a foundational reference for molecular pathogenesis three decades post-publication. Other specialized NTDs, including Leishmaniasis (Alvar et al., 2012) and Snakebite (Kasturiratne et al., 2008), also command significant influence, serving as the definitive quantitative benchmarks for their respective sub-fields in the region.

**Table 3:** Top 10 most cited Scopus-indexed articles on neglected tropical disease in Southeast Asia region

Title	Author/Year	Source	Total Citations	TC per Year	Normalized TC
The Global Distribution and Burden of Dengue	Bhatt S (2013)	Nature	7718	551.29	125.88
Global, Regional, and National Age-Sex Specific All-Cause and Cause-Specific Mortality for 240 Causes of Death, 1990–2013: A Systematic Analysis for the Global Burden of Disease Study 2013	Naghavi M (2015)	Lancet	6578	548.17	129.42
Leishmaniasis Worldwide and Global Estimates of Its Incidence	Alvar J (2012)	PLoS ONE	4279	285.27	70.28
Global, Regional, and National Age-Sex Specific Mortality for 264 Causes of Death, 1980–2016: a Systematic Analysis for the Global Burden of Disease Study 2016	Naghavi M (2017)	Lancet	4266	426.60	108.27
Global, Regional, and National Disability-Adjusted Life-Years (dalys) for 333 Diseases and Injuries and Healthy Life Expectancy (hale) for 195 Countries and Territories, 1990–2016: a Systematic Analysis for the Global Burden of Disease Study 2016	Hay SI (2017)	Lancet	1802	180.20	45.74
Schistosomiasis and Water Resources Development: Systematic Review, Meta-Analysis, and Estimates of People at Risk	Steinmman P (2006)	Lancet Infectious Diseases	1747	83.19	26.35
Dengue: a Continuing Global Threat	Guzmán MG (2010)	Nature Review Microbiology	1593	93.71	27.45
World Health Organization Global Estimates and Regional Comparisons of the Burden of Foodborne Disease in 2010	Havelaar AH (2015)	PLOS Medicine	1561	130.08	30.71
The Global Burden of Snakebite: a Literature Analysis and Modelling Based on Regional Estimates of Envenoming and Deaths	Kasturiratne A (2008)	PLOS Medicine	1502	79.05	27.38
Pathogenesis of Dengue: Challenges to Molecular Biology	Halstead SB (1988)	Science	1399	35.87	23.11

*Abbreviations: TC, total citations; Rank was based on TC

Furthermore, a clear “prestige hierarchy” is evident, as all top-cited documents were published in high-impact, Western-based multidisciplinary or medical journals (*Nature*, *The Lancet*, *Science*, *PLOS Medicine*). This presents a stark contrast to the volume-heavy regional journals identified in Table 3, which do not appear in the most influential document list. This disparity indicates that while regional journals are vital for the dissemination of primary data and surveillance, the “citation currency” of the field is heavily weighted toward Big Science collaborations and global meta-analyses published in the Global North. The high TC per year for recent GBD studies (ranging from 180 to 548) suggests that the regional research agenda is increasingly driven by data-driven modeling and large-scale systematic syntheses.

**Fig. 7:**
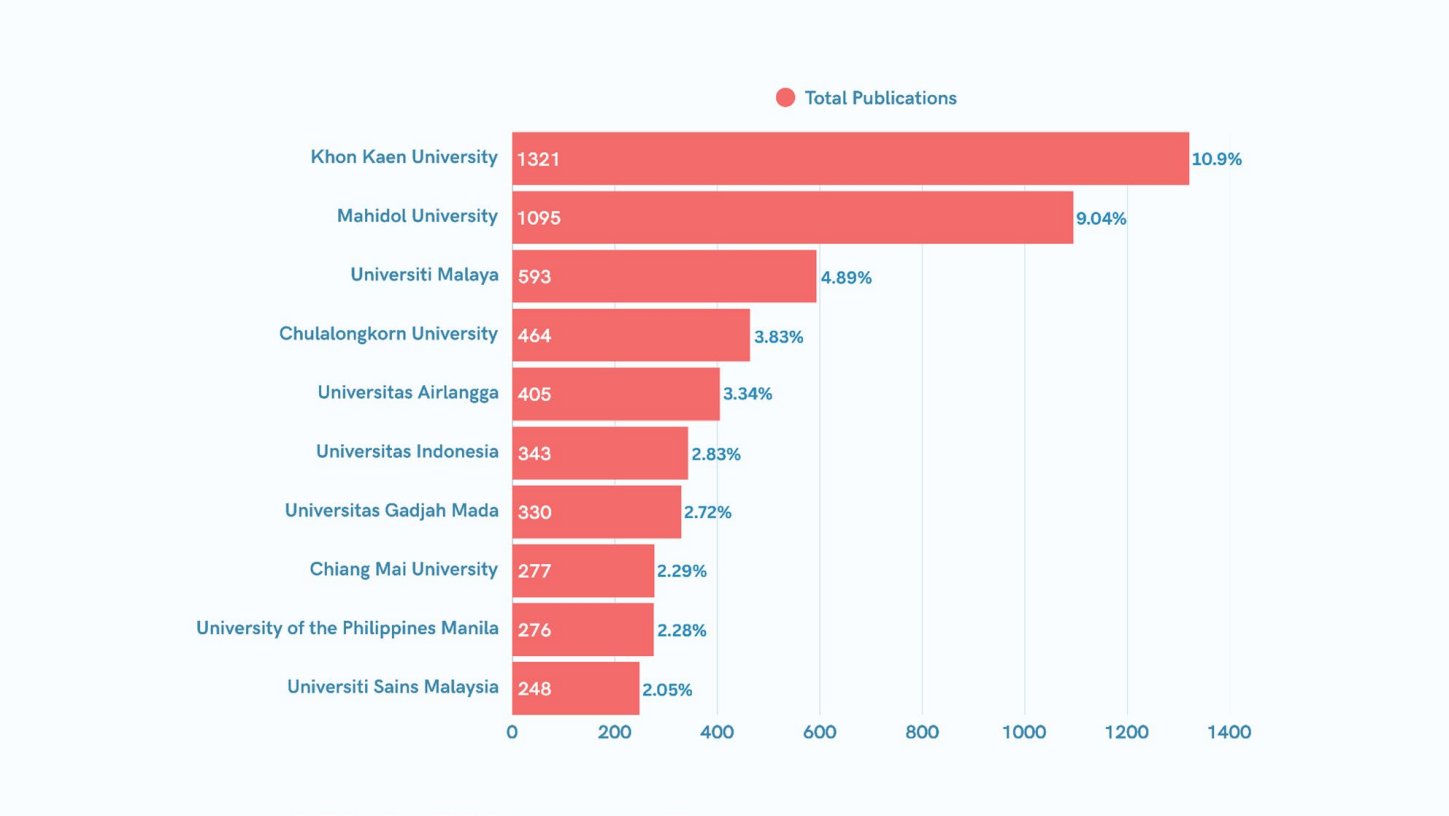
Top 10 institutions publishing NTD research in Southeast Asia

The institutional landscape of neglected tropical disease research in Southeast Asia is characterized by a significant concentration of output within a few elite Thai institutions (Fig. 7). Khon Kaen University leads the region with 1,321 publications (10.9%), followed closely by Mahidol University with 1,095 publications (9.04%). Together, these two Thai universities contribute nearly 20% of all regional research, establishing Thailand as the dominant intellectual hub for NTD studies in Southeast Asia.

Beyond the Thai leadership, the research landscape shows a secondary tier of productivity led by Malaysia and Indonesia. Universiti Malaya (4.89%) and Universiti Sains Malaysia (2.05%) represent the Malaysian contribution, while Indonesia demonstrates a broad institutional base with Universitas Airlangga (3.34%), Universitas Indonesia (2.83%), and Universitas Gadjah Mada (2.72%) all appearing in the top seven. The presence of the University of the Philippines Manila (2.28%) underscores a diverse but unevenly distributed research effort across the ASEAN member states. The sharp decline in publication volume from the 1st rank (1,321) to the 10th rank (248) suggests a “long tail” distribution, where a small number of institutions possess the specialized infrastructure and funding necessary to maintain high-volume NTD research.

This centralized model reveals a critical misalignment between institutional research capacity and the localized disease burden found in lower-income ASEAN member states. While the regional hubs drive the scientific agenda, the relatively lower output from high-burden areas in the Philippines, Vietnam, and Indonesia points to a persistent “knowledge-translation gap.” This institutional hierarchy reflects a center-periphery model that may impede the decentralization of expertise required to meet WHO 2030 NTD Road Map targets. To transition from academic excellence to equitable public health impact, the regional ecosystem requires a shift toward collaborative networks that facilitate the transfer of implementation science and diagnostic innovations from high-output hubs to the epidemiological frontlines.

### Keywords co-occurrence analysis

**Fig. 8:**
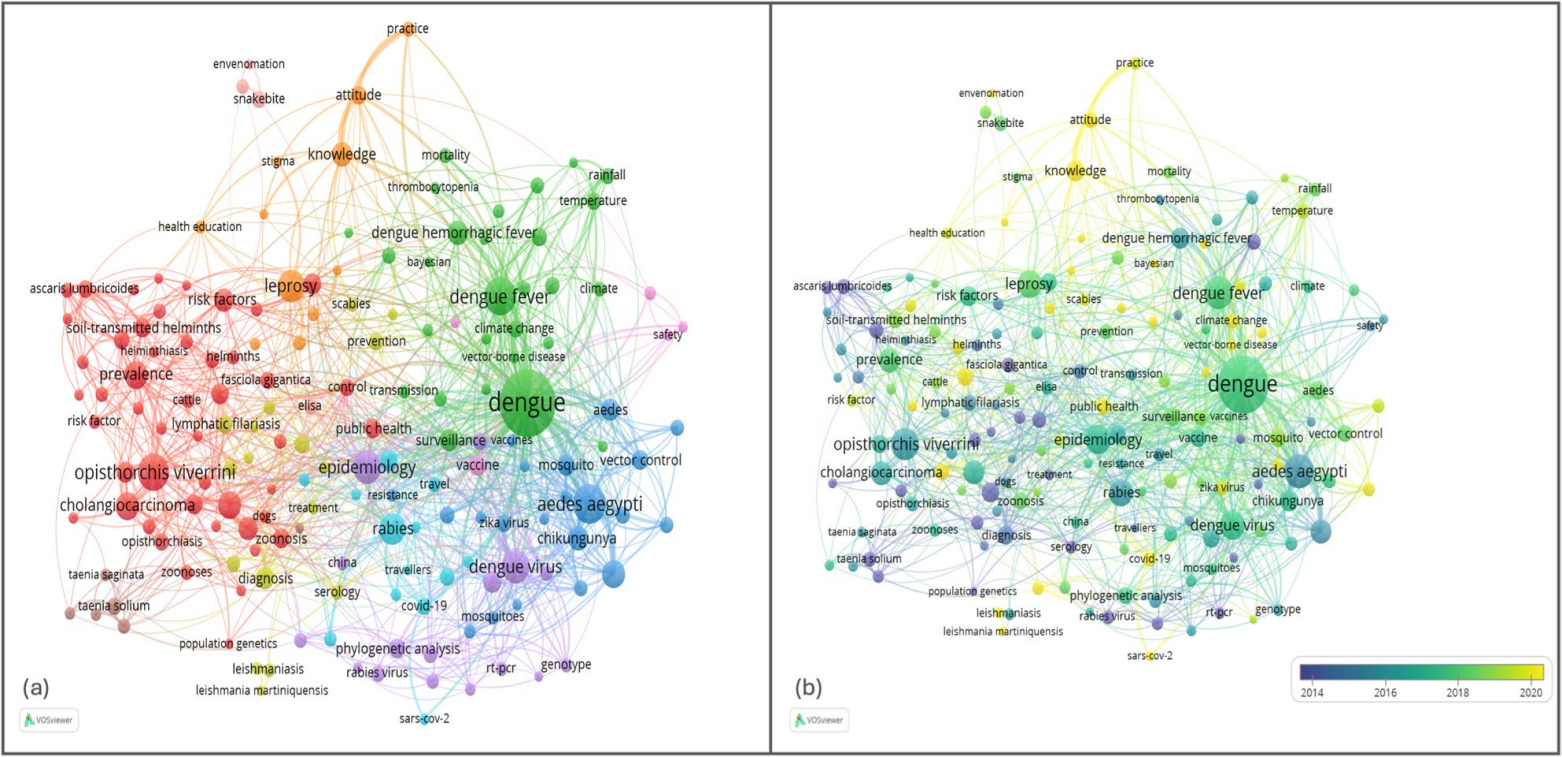
Keywords co-occurrence through (**a**) network analysis and (**b**) overlay analysis

The network analysis in Fig. 8a delineates a highly integrated research topology where “dengue” serves as the primary epistemic hub, facilitating cross-disciplinary connectivity between clinical virology, entomology, and public health. The proximity and strong link density between the central dengue cluster and the *“Aedes aegypti”* vector nodes signify a research preoccupation with the biological mechanics of transmission, while the emergence of peripheral clusters such as “knowledge, attitude, and practice” (KAP) and “cholangiocarcinoma” indicates an analytical expansion into the socio-behavioral and chronic pathological consequences of endemicity. This structure suggests that while the field remains anchored in traditional epidemiological paradigms, it increasingly functions as a multidisciplinary nexus where the convergence of socio-economic variables and co-morbid parasitic infections is being rigorously interrogated to address regional health disparities.

The overlay analysis in Fig. 8b demonstrates a significant shift from static, observational epidemiology toward dynamic, predictive modeling and global health intersectionality. The temporal transition from the blue-coded foundational studies of vector dynamics to the yellow-coded emerging frontiers of “machine learning” and “COVID-19” reflects a methodological evolution necessitated by the increasing complexity of global outbreak patterns. This progression highlights a shift in the research zeitgeist: the focus is no longer merely on describing the disease’s presence, but on leveraging computational intelligence to decouple the synergistic effects of environmental stressors, such as “climate change,” and concurrent pandemics on disease resurgence. Consequently, the visualization confirms that modern dengue research has moved into a synthetic phase, prioritizing algorithmic foresight and systemic resilience over isolated clinical interventions.

### Conceptual structure mapping

**Fig. 9:**
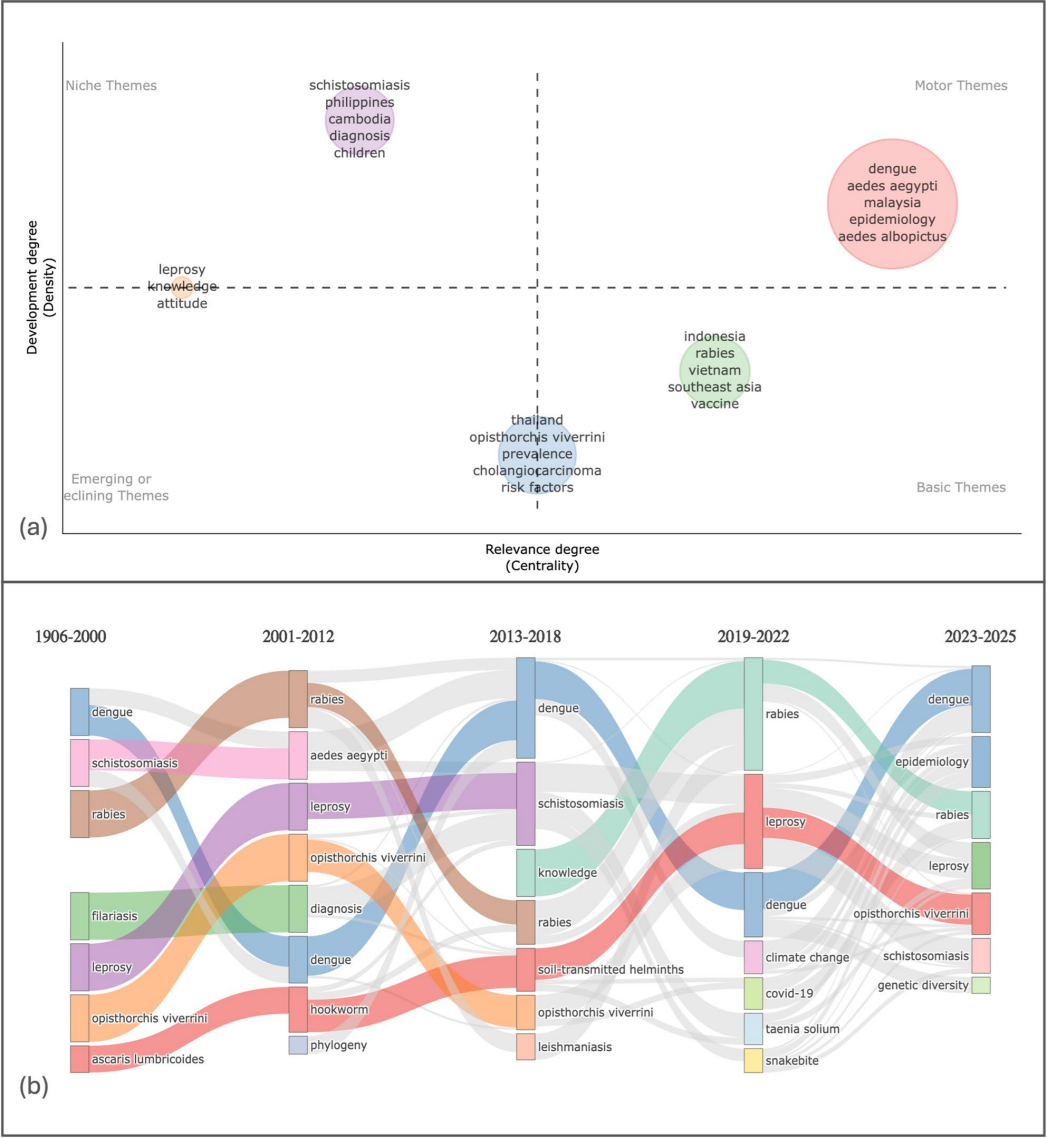
(**a**) Strategic thematic map and (**b**) temporal evolution of NTD research in Southeast Asia

The thematic map (Fig. 9a) delineates the intellectual structure of NTD research in Southeast Asia according to centrality (relevance to the overall network) and density (internal thematic cohesion). The field is anchored by a dominant motor theme centered on dengue, characterized by strong integration of vector biology (*Aedes aegypti*, *Aedes albopictus*), epidemiology, and region-specific research (notably Malaysia). Its positioning in the high-centrality/high-density quadrant indicates both conceptual maturity and structural influence, suggesting that dengue research functions as the principal organizing axis of the regional NTD knowledge system. In contrast, schistosomiasis and leprosy occupy the niche quadrant (high density but lower centrality), reflecting well-developed yet relatively specialized research trajectories—schistosomiasis emphasizing diagnosis and pediatric burden in endemic countries, and leprosy concentrating on knowledge and attitude dimensions, consistent with stigma and community-based public health frameworks. Meanwhile, themes related to rabies/vaccination (Indonesia–Vietnam) and Opisthorchis viverrine–cholangiocarcinoma (Thailand) appear as basic themes (high centrality but lower density), indicating broad relevance across the network but comparatively diffuse internal development. Collectively, the map reveals a pathogen-centered research architecture in which arboviral disease, particularly dengue, has achieved intellectual consolidation, whereas helminthic and zoonotic diseases remain important but structurally fragmented.

The thematic evolution analysis (Fig. 9b) provides a longitudinal perspective on how these structures emerged and reorganized over time. Early research (1906–2000) was dominated by classical parasitology and endemic disease documentation, with helminthic infections (e.g., filariasis, ascariasis, opisthorchiasis) and dengue forming the foundational corpus. Between 2001 and 2012, thematic branching toward vector biology, diagnostics, and phylogenetic approaches signaled methodological expansion and increasing scientific refinement. The 2013–2018 period marked consolidation around dengue and schistosomiasis, alongside the emergence of soil-transmitted helminths and behavioral constructs (e.g., knowledge), reflecting growing public health and community-oriented engagement. The 2019–2022 interval introduced diversification driven by exogenous global pressures, most notably COVID-19 and climate change, while rabies temporarily gained prominence. In the most recent period (2023–2025), the field recentralizes around dengue and epidemiology, with the appearance of genetic diversity signaling integration of molecular and genomic surveillance approaches.

The cross-sectional and longitudinal analyses demonstrate a transition from classical, geographically anchored parasitology toward increasingly data-driven, epidemiologically integrated arboviral research. Dengue has evolved from one among several endemic diseases into the structural motor theme of Southeast Asian NTD scholarship, whereas helminthic diseases persist through diversification rather than consolidation. Although the field exhibits growing methodological sophistication, evidenced by the rise of epidemiology and genetic diversity, it remains largely organized along disease-specific silos, with limited sustained integration of cross-cutting frameworks such as climate-health systems or One Health approaches. This pattern suggests both intellectual maturation and an opportunity for future systemic convergence across NTD domains in the region.

**Fig. 10:**
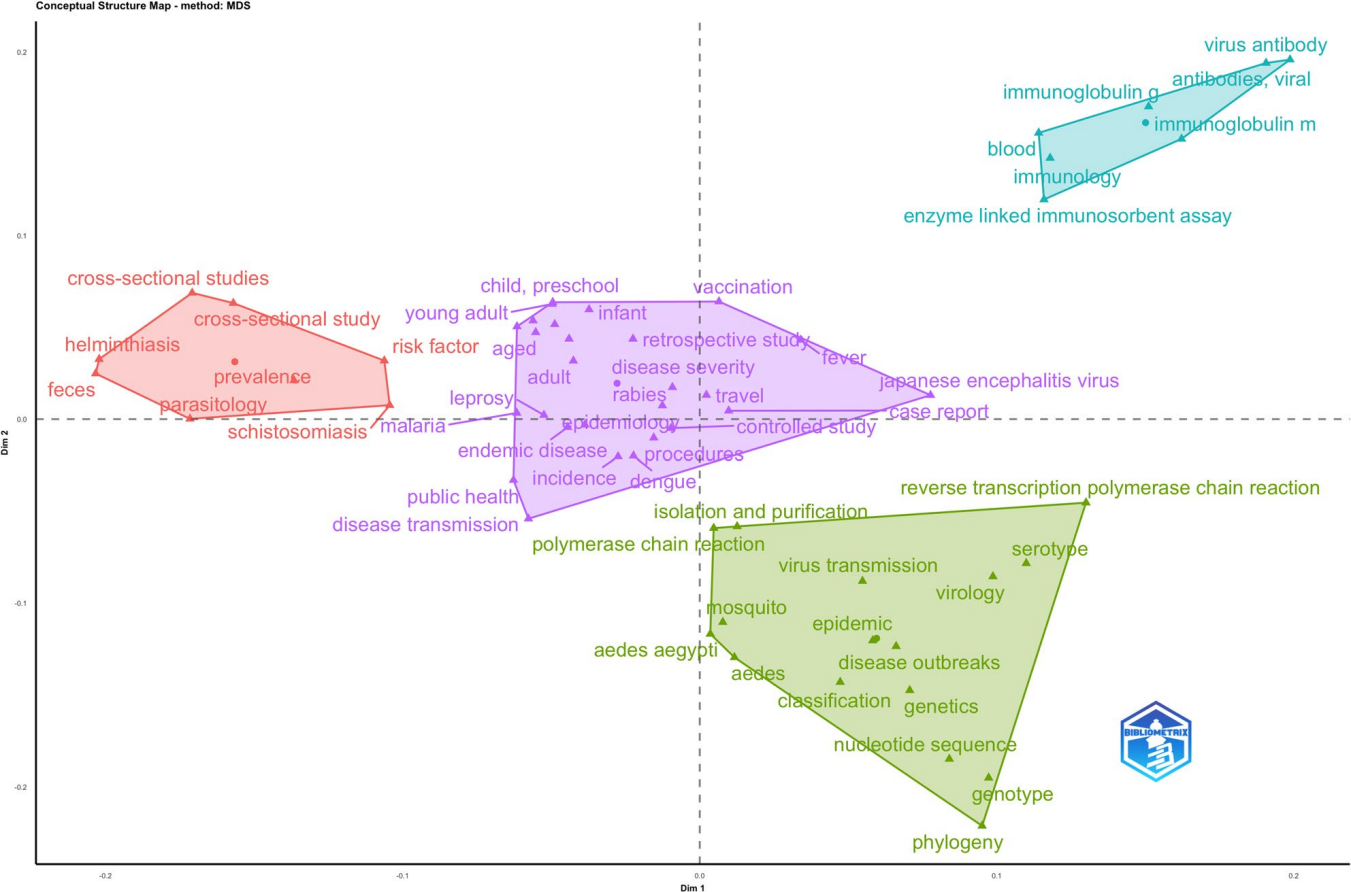
Factorial analysis through four-clustered multidimensional scaling of NTD research in Southeast Asia

The factorial map derived from multidimensional scaling (MDS) delineates the conceptual organization of neglected tropical disease research in Southeast Asia into four major thematic domains (Fig. 10), reflecting both methodological orientations and disease-specific research traditions.

At the core of the conceptual space, a centrally positioned cluster is anchored on epidemiology and public health–oriented research. This cluster is characterized by keywords related to disease transmission, endemicity, incidence, age-stratified populations, and conventional study designs (controlled, retrospective, and case-report studies). Its central location indicates that population-based surveillance, burden assessment, and transmission dynamics constitute the primary integrative framework of NTD research in the region, serving as the common reference point across otherwise heterogeneous research streams. On the negative side of Dimension 1, a distinct cluster captures classical parasitology and prevalence-focused studies, dominated by helminthic diseases such as schistosomiasis and other soil-transmitted infections. The prominence of terms such as prevalence, feces, parasitology, and cross-sectional studies suggests an emphasis on diagnostic surveys and burden estimation using traditional methodologies. The spatial separation of this cluster from molecular and immunological domains highlights a relative conceptual isolation of helminth-focused research, despite its continued epidemiological relevance in Southeast Asia.

The positive side of Dimension 1, extending into the lower quadrant, is defined by a cluster centered on molecular virology and vector-borne disease dynamics, particularly dengue-related research. This domain is structured around molecular diagnostic tools (polymerase chain reaction, reverse transcription PCR), viral genetics (genotype, phylogeny, nucleotide sequence), and vector-related concepts (*Aedes aegypti*, virus transmission, outbreaks). Its positioning indicates a methodological shift toward genomics-informed surveillance and outbreak investigation, largely distinct from classical parasitological approaches. In the upper-right quadrant, a compact cluster reflects immunological and serological research, characterized by antibody profiling, immunoglobulin subclasses, and ELISA-based diagnostics. The proximity of vaccination-related terms and viral antibody studies suggests a focus on immune response characterization and sero-epidemiological assessment. However, its relative distance from both the epidemiological core and molecular virology clusters indicates limited conceptual integration between immunological findings and broader transmission or evolutionary analyses.

This conceptual structure reveals a fragmented yet complementary research landscape of neglected tropical disease in Southeast Asia, where epidemiology functions as the central organizing axis, while parasitology, molecular virology, and immunology evolve as semi-autonomous domains. The spatial configuration among these clusters underscores the need for stronger cross-domain integration, particularly linking molecular and immunological insights with population-level epidemiology, to support operationalizing precision public health and integrated disease control strategies in resource-constrained settings.

## Discussion

This scientometric analysis provides a comprehensive perspective on the evolution, structure, and thematic orientation of neglected tropical disease (NTD) research in Southeast Asia, revealing a research ecosystem that has expanded substantially over time yet remains marked by structural and thematic imbalances. The steady growth in publication output, particularly from the early 2000s onward, reflects increased global attention to NTDs following international policy milestones such as the WHO NTD Roadmaps and the London Declaration, as well as recurrent outbreaks of vector-borne diseases across the region [1, 17]. Rather than approaching saturation, the observed logistic growth pattern suggests that NTD research in Southeast Asia has entered a phase of maturation characterized by thematic diversification and methodological refinement, consistent with trajectories observed in other globally significant infectious disease fields.

Despite this overall growth, the research landscape is highly uneven across countries. Thailand, Indonesia, Malaysia, Singapore, Vietnam, and the Philippines account for the majority of indexed publications, while countries such as Cambodia, Laos, Myanmar, Timor-Leste, and Brunei contribute minimally. This disparity mirrors broader global health research inequities, where scientific productivity is closely tied to national research infrastructure, funding availability, and integration into international collaboration networks rather than disease burden alone [18, 19]. As a result, populations experiencing substantial NTD burdens may remain underrepresented in the scholarly literature, potentially limiting the contextual relevance of evidence used to guide regional control strategies.

The thematic structure of NTD research in Southeast Asia is dominated by dengue, which consistently emerges as the central organizing theme across keyword co-occurrence networks, thematic maps, and citation analyses. This prominence reflects the epidemiological reality of dengue as one of the most pervasive and rapidly expanding vector-borne diseases in the region, driven by urbanization, climate sensitivity, and widespread Aedes mosquito distribution [20, 21]. The concentration of high-impact publications on dengue, including global burden estimates and transmission models, has elevated the disease to a motor theme that shapes the intellectual and methodological direction of the field.

While this dengue-centric focus has produced substantial scientific advances, it also exposes important limitations from an integrated disease control perspective. Other endemic NTDs—such as soil-transmitted helminthiases, schistosomiasis, food-borne trematodiases, and yaws—remain comparatively peripheral within the thematic network, despite their continued public health significance in several Southeast Asian settings. Similar imbalances have been reported in global NTD bibliometric studies, where research attention often aligns more closely with funding priorities and global visibility than with local disease burden [8, 10]. This misalignment risks perpetuating cycles of neglect for diseases that disproportionately affect marginalized and rural populations and may undermine the effectiveness of integrated control strategies that rely on addressing co-endemic conditions simultaneously.

The funding architecture provides an additional structural evidence for these thematic asymmetries. Acknowledgment analysis indicates that major financial support originates predominantly from international agencies, particularly the National Institutes of Health, the Wellcome Trust, and the Bill & Melinda Gates Foundation, alongside Japanese and Australian governmental research bodies. The alignment between these funders’ strategic priorities and the centrality of dengue, genomic surveillance, and translational infectious disease research suggests that financial capital is not merely enabling but structurally constitutive of the regional knowledge network. Although domestic funding agencies in Thailand, Malaysia, and Singapore contribute meaningfully to national research consolidation, distribution remains uneven across ASEAN member states, reinforcing infrastructural disparities and peripheral positioning of lower-capacity countries. The predominance of Global North financing thus shapes agenda-setting dynamics, privileging globally visible and security-aligned disease domains while potentially marginalizing locally burdensome but less internationally prioritized NTDs.

Encouragingly, the thematic evolution analysis indicates a gradual methodological transition from descriptive parasitology and prevalence surveys toward more predictive, data-driven approaches. The increasing prominence of mathematical modeling, spatial epidemiology, climate-linked risk analysis, and genomic surveillance reflects a broader shift toward precision public health, wherein interventions are increasingly informed by high-resolution data and real-time analytics [14, 22] These methodological advances are particularly relevant in Southeast Asia, where heterogeneous transmission patterns and rapidly changing environmental conditions demand adaptive and anticipatory control strategies.

However, despite these advances, cross-cutting frameworks central to integrated disease control, such as One Health approaches and socio-behavioral determinants of transmission, remain underdeveloped within the thematic core of the literature. Their position as emerging or peripheral themes suggests that biomedical and technological innovation has advanced more rapidly than interdisciplinary integration. This gap is notable given the recognized importance of environmental change, animal reservoirs, and human behavior in sustaining NTD transmission in the region [23, 18]. Strengthening conceptual integration across these domains will be critical for translating scientific advances into sustainable public health impact.

Collaboration patterns further illuminate the structural dynamics shaping NTD research in Southeast Asia. Co-authorship networks reveal dense collaborations between regional research hubs and institutions in high-income countries, particularly the United States, Japan, Australia, and parts of Europe. These partnerships have facilitated access to advanced technologies, funding, and high-impact publication venues, contributing to the global visibility of Southeast Asian research. At the same time, the asymmetry of these collaborations raises concerns regarding leadership, data ownership, and agenda-setting, echoing longstanding critiques of inequitable global health research practices [24, 25]. Limited intra-ASEAN collaboration suggests missed opportunities for regionally driven research agendas that are better aligned with shared epidemiological and health-system challenges.

These overall findings suggest that while NTD research in Southeast Asia has achieved significant scientific maturation, its contribution to integrated disease control remains constrained by thematic concentration, funding asymmetries, structural inequities, and limited interdisciplinary convergence. Future progress will depend not only on sustained investment in high-impact diseases such as dengue, but also on deliberate efforts to broaden research agendas to include underrepresented NTDs, strengthen South–South collaborations, diversify funding ecosystems within ASEAN, and embed One Health and socio-behavioral perspectives into core research frameworks. Aligning research production more closely with regional disease burden and health-system needs will be essential for ensuring that the expanding evidence base meaningfully supports the goals of the WHO NTD Roadmap 2021–2030 and advances equitable, integrated disease control strategies across Southeast Asia.

## Conclusion

The NTD research landscape in Southeast Asia has evolved into a mature yet structurally imbalanced domain, characterized by robust aggregate growth masked by persistent inequities in research capacity and thematic specialization. While the overwhelming concentration on dengue reflects immediate epidemiological pressures, it simultaneously exposes a “neglect within the neglected”—the systemic marginalization of diseases such as yaws, Buruli ulcer, and foodborne trematodiases that lack equivalent commercial or political visibility. The superior research performance of Thailand, Indonesia, and Malaysia establishes a potent “regional core,” yet its sharp contrast with the limited output of lower-income neighbors highlights enduring disparities that decouple knowledge production from the areas of highest relative disease burden. The observed transition toward interdisciplinary, data-driven paradigms, specifically spatial epidemiology and genomic analytics, presents transformative opportunities for precision public health. However, the benefits of these scientific dividends will remain unevenly distributed without structural reforms to strengthen national research systems and foster intra-regional cooperation. Currently, the predominance of extra-regional collaboration networks, coupled with sparse intra-ASEAN scientific exchange, suggests that regional knowledge integration remains underdeveloped despite shared eco-epidemiological challenges. Advancing the mandates of the WHO 2030 NTD Roadmap necessitates a structural recalibration of the regional research ecosystem. Critical priorities include incentivizing South-South collaborations, institutionalizing One Health frameworks, and democratizing access to genomic and digital surveillance platforms to reduce epistemic inequities. Ultimately, a teleological shift toward inclusive, regionally coordinated, and burden-aligned research agendas is essential to generate the context-sensitive evidence required for integrated, climate-resilient, and precision-informed NTD control across Southeast Asia.

## Data Availability

The data used in this study are present in the manuscript.

## Appendix I

Search string: ( TITLE-ABS-KEY( “neglected tropical disease*” OR “NTD*” OR dengue OR chikungunya OR “buruli ulcer” OR “mycobacterium ulcerans” OR echinococcosis OR “echinococcus granulosus” OR “echinococcus multilocularis” OR “foodborne trematode*” OR clonorchis OR opisthorchis OR fasciola OR paragonimus OR leishmaniasis OR leishmania OR leprosy OR “hansen* disease” OR “mycobacterium leprae” OR “lymphatic filariasis” OR filariasis OR elephantiasis OR hydrocele OR mycetoma OR actinomycetoma OR eumycetoma OR chromoblastomycosis OR noma OR “cancrum oris” OR rabies OR scabies OR ectoparasitoses OR “sarcoptes scabiei” OR schistosomiasis OR bilharzia OR schistosoma OR “soil-transmitted helminth*” OR ascaris OR trichuris OR hookworm OR “necator americanus” OR “ancylostoma duodenale” OR “snakebite envenoming” OR snakebite OR taeniasis OR cysticercosis OR “taenia solium” OR “taenia saginata” OR “taenia asiatica” OR trachoma OR yaws OR “endemic treponematoses” ))AND( TITLE-ABS-KEY( “Southeast Asia” OR “South-East Asia” OR ASEAN OR Brunei OR Cambodia OR Indonesia OR Laos OR “Lao PDR” OR Malaysia OR Myanmar OR Philippines OR Singapore OR Thailand OR Vietnam OR “Timor-Leste” )) AND ( EXCLUDE ( PUBYEAR,“2026” ) ) AND ( LIMIT-TO ( LANGUAGE,“English” ) ) AND ( LIMIT-TO ( DOCTYPE,“re” ) OR LIMIT-TO ( DOCTYPE,“ar” )).
